# Differences in Oral Sexual Behaviors by Gender, Age, and Race Explain Observed Differences in Prevalence of Oral Human Papillomavirus Infection

**DOI:** 10.1371/journal.pone.0086023

**Published:** 2014-01-24

**Authors:** Gypsyamber D’Souza, Kevin Cullen, Janice Bowie, Roland Thorpe, Carole Fakhry

**Affiliations:** 1 Department of Epidemiology, Johns Hopkins Bloomberg School of Public Health, Baltimore, Maryland, United States of America; 2 Department of Otolaryngology-Head and Neck Surgery, Johns Hopkins Hospital, Baltimore, Maryland, United States of America; 3 Greenebaum Cancer Center, University of Maryland, Baltimore, Maryland, United States of America; 4 Department of Health, Behavior and Society, Johns Hopkins Bloomberg School of Public Health, Baltimore, Maryland, United States of America; 5 Department of Health Policy and Management, Johns Hopkins Bloomberg School of Public Health, Baltimore, Maryland, United States of America; 6 Hopkins Center for Health Disparities Solutions, Johns Hopkins Bloomberg School of Public Health, Baltimore, Maryland, United States of America; 7 Milton J. Dance Jr. Head and Neck Cancer Center, Greater Baltimore Medical Center, Baltimore, Maryland, United States of America; Georgetown University, United States of America

## Abstract

**Purpose:**

This study explores whether gender, age and race differences in oral sexual behavior account for the demographic distribution of oral human papillomavirus infection (HPV) and HPV-positive oropharyngeal cancer (HPV-OSCC)

**Methods:**

This analysis included 2,116 men and 2,140 women from NHANES (2009–10) who answered a behavioral questionnaire and provided an oral-rinse sample for HPV detection. Weighted prevalence estimates and prevalence ratios (PR) were calculated for sexual behaviors and oral HPV infection by gender, age-cohort (20–29, 30–44, 45–59, 60–69), and race, and contrasted with incidence rate ratios (IRR) of OSCC from SEER 2009. Multivariate logistic regression was used to evaluate predictors of oral sexual behavior and oral HPV16 infection.

**Results:**

Differences in oral sexual behavior were observed by gender, age-cohort and race. Most men (85.4%) and women (83.2%) had ever performed oral sex, but men had more lifetime oral and vaginal sexual partners and higher oral HPV16 prevalence than women (each p<0.001). 60–69 year olds (yo) were less likely than 45–59 or 30–44 (yo) to have performed oral sex (72.7%, 84.8%, and 90.3%, p<0.001), although oral HPV16 prevalence was similar. Prevalence ratios (PR) of ever oral sex in men vs. women (PR = 1.03), and 45–59 vs. 30–44 year-old men (PR = 0.96) were modest relative to ratios for oral HPV16 infection (PRs = 1.3–6.8) and OSCC (IRR = 4.7–8.1). In multivariate analysis, gender, age-cohort, and race were significant predictors of oral sexual behavior. Oral sexual behavior was the primary predictor of oral HPV16 infection; once this behavior was adjusted for, age-cohort and race were no longer associated with oral HPV16.

**Conclusion:**

There are differences in oral sexual behaviors when considering gender, age-cohort and race which explain observed epidemiologic differences in oral HPV16 infection across these groups.

## Background

Human papillomavirus (HPV), a sexually transmitted infection, causes a subset of oropharyngeal squamous cell cancers (OSCCs). [1] From 1973 to 2004, the incidence of HPV-positive OSCC (HPV-OSCC) in the United States (U.S.) rose steadily, while the incidence of HPV-negative OSCC (HPVn-OSCC) decreased. [2], [3] The decline in HPVn-OSCC has largely been attributed to the success of anti-smoking campaigns. [4] By contrast, the recent rise in HPV-OSCC has been speculated to be a result of the sexual revolution coupled with the maturation of the so-called “baby boom” generation [2], [5].

In recent decades sexual behaviors have changed; the age of sexual initiation has decreased, and the lifetime number of sexual partners has increased. [6]–[12] In addition, ever having performed oral sex varies with age and may differ by race, [12] with oral sex being more prevalent among whites (∼75%) than blacks (∼62%) or Hispanics (∼63%). [13], [14] Interestingly, the increase in HPV-OSCC incidence in the U.S. is observed among men and whites, but not among women or blacks. [2] Indeed, individuals with HPV-OSCC have a distinct demographic profile, tending to be male, younger, and of higher socioeconomic status than those with HPVn-OSCC. [1], [15]–[18] Studies consistently report that a significantly higher proportion of white (21–64%) than black (0–35%) OSCC cases are HPV-positive. [15]–[19] The lower proportion of HPV-OSCC among blacks might be explained by a higher incidence of HPVn-OSCC and/or lower rates of HPV-OSCC among blacks than whites [16], [19]–[21].

Similar to the differences seen in HPV-OSCC rates, oral HPV prevalence in the general population varies by gender, age, and race. [22] Presently, it is unclear whether differences in sexual behavior of men, younger age cohorts, and whites, explain the higher oral HPV infection and HPV-OSCC rates in these groups. Therefore, we examined differences in sexual behaviors by gender, age-cohort, and race in a nationally representative sample, to explore whether the observed epidemiologic differences in oral HPV infection and OSCC rates reflect differences in sexual behaviors across these groups.

## Methods

Data from the 2009–10 National Health and Nutrition Examination Survey (NHANES) were used in this analysis. NHANES is a stratified multistage probability sample of the U.S. population, that oversamples blacks and Hispanics, and people ≥60 yo [23].

### Behavioral Data

Demographic data was collected using an interviewer administered survey. Race and ethnicity were self-identified by participants and reported by NHANES as: Mexican-American, other Hispanic, non-Hispanic white, non-Hispanic black, and other race including multi-racial [23].

Sexual behavioral data were collected by audio computer-assisted interview (ACASI). Variables included ever having had any sex, ever performing oral sex (referred to as “oral sex” hereafter), age at first sex, and age first performed oral sex. Age at first oral sex was compared to age at first sexual act (referred to as “sexual debut” hereafter); we considered first oral sex to be “at” sexual debut if age at first oral sex was the same as age at first sex. Number of lifetime partners for any kind of sex, vaginal sex, and performing oral sex, were each collected separately. Number of lifetime partners reported in this paper is a sum of both male and female partners. Participants ≥60 yo were not asked about lifetime number of oral sex partners therefore analysis for this variable was restricted to those between 20–59 yo. Women ≥60 yo were not asked about number of female (i.e. lesbian) sexual partners. Subjects <20 years of age were excluded because of more limited sexual data in this group and because their current sexual experience does not reflect their expected cumulative exposure.

### Oral Rinse Samples and HPV DNA Detection

Oral rinse sample collection and HPV detection (Roche Linear Array HPV Genotyping Test) are described on the NHANES website and by Gillison et al. [22], [23] Individuals positive for any of the 37 HPV types were considered to have a prevalent oral HPV infection. [22], [24] As more than 90% of HPV-OSCCs are caused by HPV16, oral HPV16 prevalence was also considered separately.

### Statistical Analysis

Analysis for this study was restricted to individuals 20–69 yo, as the ACASI was not administered to older individuals. 5,001 individuals of this age completed any part of the 2009–10 ACASI, however, analysis was restricted to the 4,256 (85%) who answered the sexual behavior questions.

Weighted prevalence estimates were reported. The primary outcomes of interest were history of ever performing oral sex and number of lifetime oral sex partners. These outcomes were stratified by gender, age-cohort, and race/ethnicity. Age categories were chosen a-priori to represent generational cohorts who had expected peak sexual exposure around the 1960s, 1970–85, 1986–99 & 2000–10; subjects were divided by age into these corresponding strata of 60–69 yo (called “seniors” hereafter), 45–59 yo (“middle-aged”), 30–44 yo (“adults”), and 20–29 yo (“young adults”). To account for the complex NHANES survey design, all analyses used NHANES 2009–2010 MEC sample weights, and thus provided unbiased estimates of behavior and oral HPV prevalence for the civilian, non-institutionalized U.S. population. Variances were computed with SUDAAN software version 10.0.1 (RTI, Research Triangle Park, North Carolina) using the Taylor series linearization method. Wald F p-values were used to compare weighted prevalence between groups. For continuous variables, weighted means were compared using analysis of variance with Wald F p-values.

The association of gender, age-cohort and race with odds of ever having performed oral sex was explored using multivariate logistic regression. Multivariate risk factors for any prevalent oral HPV and with oral HPV16 infection were each similarly modeled. These models were adjusted for potential confounders including: any college education, ever married, ever smoked cigarettes (>100 cigarettes in lifetime), and ever drank alcohol regularly (≥1 drink/month), each yes/no; models for HPV also adjusted for either ever oral sex (among all subjects), or for number of oral sex partners (among 20–59 yo). With the exception of age-cohort, gender, and race, only covariates that were statistically significant were retained in the final multivariate models. Multivariate associations with number of lifetime oral sex partners were similarly explored using linear regression, restricting to individuals <60 years olds since this data was not collected among older individuals. Prevalence ratios (PR) were calculated for oral sexual behaviors of interest and oral HPV16 infection.

Age-adjusted incidence rate ratios (IRR) of OSCC were similarly compared by gender, age-cohort, and race, using data from the 2009–10 Surveillance, Epidemiology and End Results (SEER) Program [25] for 20–69 year-olds. OSCC was defined in this SEER analysis to include cancers with following ICD-O codes: C01.9 [BOT], C02.4 [lingual tonsil], C05.1 (soft palate NOS), and C05.2 (uvula), C09.0-C09.9 [tonsil], C10.0 [vallecula ], C10.2-C10.9 [OP], C14.0 [pharynx NOS], and C14.2 [waldeyers ring].

## Results

Data from 2,116 men and 2,140 women between the ages of 20 and 69 were included in this analysis (Table S1 in File S1). Participants were primarily heterosexual (92.5%), and half had more than five lifetime sexual partners (50.3%).

### Differences in Behavior by Gender

Sexual behaviors of interest were first compared by gender (Table 1). The overwhelming majority of men (85.4%) and women (83.2%) had performed oral sex. Men were more likely than women to have >5 lifetime sexual (59.7% vs. 41.0%, p<0.001), or oral sex (32.4% vs. 17.6%, p<0.001) partners. Similarly, prevalence of oral HPV16 (2.0% vs. 0.3%, p<0.001) and any oral HPV infection (11.4% vs. 3.3%, p<0.001) was significantly higher among men than women. Prevalence of any oral HPV infection was similar among 52 gay/bisexual men and 1549 heterosexual men (13.3% vs. 11.0%, p = 0.65) and higher but not significantly different in 93 lesbian/bisexual women compared with 1504 heterosexual women (6.1% vs. 3.2%, p = 0.45).

**Table 1:** Sexual behavior and oral HPV prevalence, overall and gender stratified.

	Overall	Male	Female	
		(N = 2,116)	(N = 2,140)	P-value
**Number of lifetime sexual partners:**				
Any sexual act: median	6	8	5	–
Any sexual act: mean	13.2	18.4	7.8	<0.001
Performed oral sex on*: median	2	3	2	–
Performed oral sex on*: mean	6.9	9.9	3.8	0.001
**Age at first:**				
Sexual act: median	17	17	17	–
Sexual act: mean	17.5	17.3	17.7	0.031
Performed oral sex on*: median	19	18	19	–
Performed oral sex on*: mean	19.8	19.5	20.2	0.002
**Number of lifetime sexual partners**				<0.001
0	3.3%	3.9%	2.8%	
1–2	21.5%	17.2%	25.9%	
3–5	24.7%	19.2%	30.3%	
6–10	23.1%	22.3%	23.9%	
>10	27.4%	37.4%	17.1%	
**Ever performed oral sex**				0.018
No	15.7%	14.6%	16.8%	
Yes	84.3%	85.4%	83.2%	
**Number of people performed oral sex on in lifetime** *				<0.001
0	13.8%	12.9%	14.8%	
1–2	36.2%	29.8%	42.8%	
3–5	24.9%	24.9%	24.9%	
6–10	13.3%	14.8%	11.7%	
>10	11.8%	17.6%	5.9%	
**Comparison of number of lifetime oral and vaginal sexual partners** *				0.008
Fewer oral sex partners	64.7%	62.4%	67.0%	
Same # oral & vaginal	24.8%	26.5%	23.1%	
More oral sex partners	10.5%	11.1%	9.9%	
**First sexual experience** <18 yo	54.9%	56.9%	52.9%	0.031
**First performed oral sex** * <18 yo	29.6%	32.1%	27.0%	0.035
**Age at first oral sex relative to sexual debut** *				0.092
Oral sex at sexual debut	35.4%	37.0%	33.7%	
Oral sex after sexual debut	50.8%	50.1%	51.5%	
Never oral sex	13.8%	12.8%	14.8%	
**Oral HPV prevalence**	N = 4059	N = 2030	N = 2029	
HPV16	1.1%	2.0%	0.3%	<0.001
Any HPV type	7.4%	11.4%	3.3%	<0.001

* These categories do not include data on 60–69 year old individuals since this data was not collected in that age group.

### Differences in Behavior by Age-cohort

Compared to younger age-cohorts, seniors (60–69 yo) had the oldest age at sexual debut (mean = 18.8 years) and were less likely to have ever performed oral sex (72.7%), or to have had >5 sexual partners (39.4%) (each p<0.001, Table S2 in File S1). Middle aged individuals (45–59 yo) were significantly less likely than adults (30–44 yo) to report ever performing oral sex (84.8% vs. 90.3%), >5 lifetime oral sexual partners (25.5% vs. 28.5%), first oral sex before age 18 (19.5% vs. 30.9%), and oral sex at/around sexual debut (26.5% vs. 38.5%), (each p<0.05, Table S2 in File S1).

**Figure 1:**
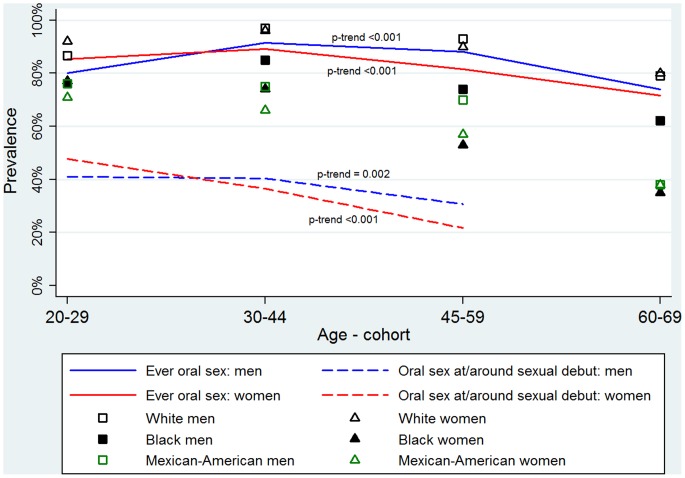
Prevalence of ever performing oral sex and performing oral sex at/around sexual debut, by gender, age-cohort and race/ethnicity groups.

Given the behavioral differences observed between men and women, additional age-cohort analyses were stratified by gender (Table 2). Among both genders, older age-cohorts had fewer lifetime sexual partners and an older age of sexual debut (each p≤0.02). While the majority of individuals in each age-cohort, including seniors, reported ever performing oral sex, seniors were significantly less likely to have ever performed oral sex than adults (males 73.9% vs. 91.4%; females 71.6% vs. 89.1%, each p<0.001) (Table 2, Figure 1). Oral HPV and oral HPV16 prevalence did not differ significantly across age-cohorts for both genders (Table 2).

**Table 2:** Sexual behavior and oral HPV prevalence, by age-cohort and gender.

	Males (N = 2,116)					Females (N = 2,140)				
	Age in years					Age in years				
	20–29	30–44	45–59	60–69		20–29	30–44	45–59	60–69	
	(young adult)	(adult)	(middle age)	(senior)		(young adult)	(adult)	(middle age)	(senior)	
	N = 417	N = 646	N = 654	N = 399	P- value	N = 470	N = 653	N = 622	N = 395	P- value
**Number of lifetime sexual partners:**										
Any sexual act: median	5	9	9	6	–	5	5	5	3	–
Any sexual act: mean	10.5	19.3	21.5	21.5	<0.001	8.4	8.5	7.9	5.6	0.024
Performed oral sex on*: median	2	4	3	–	–	2	2	2	–	–
Performed oral sex on*: mean	4.7	10.6	12.8	–	<0.001	4.1	4.1	3.3	–	0.031
**Age at first:**										
Sexual act: median	16	16	17	18	–	16	17	18	18	–
Sexual act: mean	16.4	17.2	17.6	18.5	<0.001	16.4	17.4	18.1	19.2	<0.001
Performed oral sex on*: median	17	18	19	–	–	17	19	20	–	–
Performed oral sex on*: mean	17.7	19.4	20.7	–	<0.001	17.5	20.1	22.0	–	<0.001
**Number of lifetime sexual partners**					<0.001					<0.001
0	10.2%	1.8%	1.8%	3.3%		7.4%	1.1%	2.1%	1.5%	
1–2	19.4%	14.2%	16.5%	22.0%		21.2%	23.3%	23.6%	43.1%	
3–5	21.3%	18.9%	17.9%	19.9%		24.7%	27.4%	36.6%	30.3%	
6–10	23.1%	22.9%	23.0%	17.8%		24.9%	30.5%	21.1%	14.9%	
>10	26.0%	42.1%	40.7%	37.1%		21.8%	17.8%	16.6%	10.1%	
**Ever performed oral sex**					<0.001					<0.001
No	19.9%	8.6%	12.0%	26.1%		14.6%	10.9%	18.4%	28.4%	
Yes	80.1%	91.4%	88.0%	73.9%		85.4%	89.1%	81.6%	71.6%	
**Number of people performed oral sex on in lifetime** *					<0.001					0.026
0	20.0%	8.6%	12.2%	–		14.7%	11.0%	18.4%	–	
1–2	32.0%	27.7%	30.4%	–		37.7%	45.1%	44.0%	–	
3–5	27.5%	26.0%	22.1%	–		28.8%	25.0%	22.1%	–	
6–10	11.1%	15.9%	16.2%	–		10.9%	12.5%	11.4%	–	
>10	9.3%	21.8%	19.1%	–		7.8%	6.4%	4.1%	–	
**Comparison of number of lifetime oral and vaginal sexual partners** *					0.273					0.115
Fewer oral sex partners	58.5%	63.7%	63.8%	–		59.9%	66.5%	72.1%	–	
Same # oral & vaginal	27.6%	26.3%	26.0%	–		26.3%	22.6%	21.5%	–	
More oral sex partners	13.9%	10.0%	10.2%	–		13.8%	10.9%	6.5%	–	
**First sexual experience** <18 years	61.6%	63.5%	54.1%	40.7%	<0.001	64.4%	59.3%	48.1%	34.1%	<0.001
**First performed oral sex** <18 years*	41.4%	34.8%	23.2%	–	<0.001	44.3%	26.8%	15.7%	–	<0.001
**Age at first oral sex relative to sexual debut** *					0.002					<0.001
Oral sex at sexual debut	41.0%	40.4%	31.1%	–		47.9%	36.5%	21.8%	–	
Oral sex after sexual debut	39.0%	51.0%	56.8%	–		37.5%	52.5%	59.8%	–	
Never oral sex	19.9%	8.6%	12.1%	–		14.7%	10.9%	18.5%	–	
**Oral HPV prevalence**	N = 407	N = 609	N = 627	N = 387		N = 459	N = 619	N = 581	N = 372	
HPV16	1.5%	1.8%	2.3%	2.1%	0.929	0.2%	0.1%	0.5%	0.5%	0.153
Any HPV type	8.2%	10.4%	13.5%	13.5%	0.292	4.0%	2.3%	3.7%	3.5%	0.581

* These categories do not include data on 60–69 year old individuals since this data was not collected in that age group.

For both genders and all age-cohorts, the majority of individuals had fewer lifetime oral than vaginal sexual partners. However, young men and women were more likely than older age groups to have oral sex at/around sexual debut (each p<0.01, Table 2).

### Differences in Behavior by Race

Given the observed differences in prevalence of oral HPV16 infection and incidence of HPV-OSCC among racial groups in the U.S., we also evaluated the prevalence of sexual risk factors by race/ethnicity. The number of lifetime sexual and oral sex partners and age of first sex varied considerably between racial and ethnic groups (Table 3). Across all age-cohorts white men and women were more likely than individuals of other races and ethnicities to have ever performed oral sex (each p<0.001, Figure 1).

**Table 3:** Sexual behavior and oral HPV prevalence, by race/ethnicity∼ and gender.

	Male (N = 2,116)						Female (N = 2,140)					
	White	Black	Mexican-American	Hispanic	Other	P- value	White	Black	Mexican-American	Hispanic	Other	P- value
	N = 957	N = 404	N = 429	N = 224	N = 102		N = 985	N = 377	N = 408	N = 258	N = 112	
**Number of lifetime sexual partners:**												
Any sexual act: median	7	12	5	10	2	–	5	6	2	3	3	–
Any sexual act: mean	18.4	26.6	15.7	15.6	10.8	0.024	8.3	9.3	5.0	5.4	5.5	<0.001
Performed oral sex on*: median	4	3	2	3	1	–	2	2	1	2	1	–
Performed oral sex on*: mean	12.6	5.2	4.2	6.2	3.1	<0.001	4.5	2.5	2.1	2.7	2.6	<0.001
**Age at first:**												
Sexual act: median	17	15	17	16	19	–	17	16	17	18	19	–
Sexual act: mean	17.5	15.5	17.1	15.8	20.7	<0.001	17.6	16.7	17.9	18.0	19.6	0.003
Performed oral sex on*: median	18	19	20	19	20	–	18	20	20	20	20	–
Performed oral sex on*: mean	19.0	20.4	21.0	19.1	22.3	<0.001	19.6	21.9	21.2	21.9	21.5	<0.001
**Number of lifetime sexual partners**						<0.001						<0.001
0	2.4%	4.4%	7.4%	3.9%	12.8%		1.7%	3.5%	5.8%	5.0%	7.9%	
1–2	17.9%	4.8%	16.2%	9.3%	39.3%		23.6%	13.1%	50.6%	34.4%	36.5%	
3–5	19.7%	12.7%	26.3%	17.6%	15.1%		30.9%	31.8%	23.8%	33.5%	25.5%	
6–10	22.8%	22.9%	23.5%	22.1%	14.3%		24.7%	33.0%	9.9%	17.9%	20.3%	
>10	37.2%	55.3%	26.6%	47.1%	18.5%		19.0%	18.6%	9.9%	9.3%	9.8%	
**Ever performed oral sex**						<0.001						<0.001
No	9.2%	23.5%	28.0%	15.4%	37.1%		9.3%	36.8%	36.9%	24.5%	26.1%	
Yes	90.8%	76.5%	72.0%	84.6%	62.9%		90.7%	63.2%	63.1%	75.5%	73.9%	
**Number of people performed oral sex on in lifetime** *						<0.001						<0.001
0	7.0%	21.8%	26.0%	14.1%	34.4%		7.0%	32.8%	34.7%	22.3%	23.9%	
1–2	28.8%	25.8%	37.7%	26.6%	36.1%		43.2%	36.2%	46.2%	44.8%	44.7%	
3–5	25.3%	31.7%	21.9%	25.5%	13.8%		27.4%	22.8%	10.9%	26.8%	20.5%	
6–10	18.0%	9.1%	5.0%	17.4%	5.8%		15.3%	5.8%	4.4%	4.2%	4.4%	
>10	20.8%	11.6%	9.4%	16.4%	9.9%		7.1%	2.3%	3.9%	1.9%	6.5%	
**Comparison of number of lifetime oral and** **vaginal sexual partners** *						<0.001						<0.001
Fewer oral sex partners	59.2%	79.7%	67.0%	72.4%	49.3%		66.4%	82.0%	62.7%	64.8%	53.5%	
Same # oral & vaginal	28.6%	12.4%	21.7%	19.3%	43.8%		22.6%	9.1%	31.1%	28.0%	38.4%	
More oral sex partners	12.2%	7.9%	11.3%	8.3%	7.0%		11.1%	8.9%	6.2%	7.2%	8.1%	
**First sexual experience** <18 years	55.7%	75.4%	56.8%	72.3%	25.6%	<0.001	53.8%	66.6%	48.0%	46.0%	30.2%	<0.001
**First performed oral sex** * <18 years	37.5%	23.0%	22.4%	27.2%	13.0%	<0.001	32.8%	14.4%	14.7%	16.2%	19.2%	<0.001
**Age at first oral sex relative to sexual debut** *												
Oral sex at sexual debut	43.5%	17.6%	25.9%	26.5%	32.0%	<0.001	40.5%	12.7%	23.1%	21.2%	31.7%	<0.001
Oral sex after sexual debut	49.5%	60.7%	48.2%	59.4%	33.4%		52.5%	54.6%	42.1%	56.5%	44.3%	
Never oral sex	7.0%	21.7%	25.8%	14.1%	34.6%		7.0%	32.7%	34.7%	22.3%	23.9%	
**Oral HPV prevalence**	**N = 899**	**N = 396**	**N = 416**	**N = 221**	**N = 98**		**N = 923**	**N = 365**	**N = 386**	**N = 246**	**N = 109**	
HPV16	2.4%	1.9%	0.8%	1.3%	0.0%	<0.001	0.4%	0.0%	0.0%	0.3%	0.0%	0.152
Any HPV type	10.8%	20.1%	8.1%	10.3%	9.0%	0.027	3.2%	3.5%	4.0%	4.5%	2.2%	0.696

∼ Race/ethnicity categories included white non-Hispanic (“white”), black non-Hispanic (“black”), Mexican-American, black and white Hispanic (“Hispanic”), and other races/multi-race (“other”).

* These categories do not include data on 60–69 year old individuals since this data was not collected in that age group.

White men had the highest number of lifetime oral sex partners (mean = 12.6), and the youngest age of first performing oral sex (mean = 19.0 yo) compared to all other racial/ethnic groups (Table 3, p<0.001). By contrast, black men had the highest number of lifetime sexual partners for any sexual act (mean = 26.6, p = 0.024) and the youngest age of first sexual act (mean = 15.5 yo, p<0.001). White men were significantly more likely than black men to have >5 lifetime oral sex partners (38.8% vs. 20.7%, p<0.001) and to have performed oral sex at/around sexual debut (43.5% vs. 17.6%, p<0.001). A lower proportion of men reporting >5 lifetime oral sex partners was also observed among Mexican-American men (14.4%, p<0.001) and men of other race (15.7%, p = 0.002), although not among other Hispanic men (33.8%, p = 0.23) compared to white men. Similar differences were observed between white and black women (Table 3), and when comparing white women with Mexican-American, Hispanic, women of other race (Table 3).

Reporting fewer lifetime oral than vaginal sexual partners was significantly more common among blacks than whites (men 79.7% vs. 59.2%; women 82.0% vs. 66.4%, each p<0.001). Similar differences were observed when comparing whites with other racial/ethnic groups (Table 3).

### Relative Differences in Oral HPV Infection and Oropharyngeal Cancer

The behavioral differences observed by gender, age-cohort, and race are consistent with changing HPV-OSCC trends; i.e. males, younger age-cohorts, and whites were significantly more likely to report oral sexual behaviors of interest. To understand whether these behavioral differences could explain the observed demographic differences in oral HPV16 infection and HPV-OSCC incidence, we compared the prevalence ratios of oral sexual behaviors and oral HPV16 infection, with SEER incidence rate ratios of OSCC across these groups of interest (Table 4).

**Table 4:** Prevalence ratios (PR) of sexual behavior*, and oral HPV infection and incidence rate ratios (IRR) of oropharyngeal cancer diagnosis, by gender, age-cohort and race.

	NHANES DATA FROM 2009–2010				SEER DATA FROM 2009
	Sexual behavior				
	Ever performedoral sex	5 lifetime oralsex partners*	Performed oralsex at/aroundfirst sexual act*	Oral HPV16Infection	Age adjusted OSCC incidence/100,000
**Gender**					
Male	85.4%	32.4%	37.0%	1.95%	9.1
Female	83.2%	17.6%	33.7%	0.29%	1.9
Ratio** (M/F)	**1.03 (1.01,1.05)**	**1.84 (1.54, 2.20)**	**1.10 (0.99, 1.22)**	**6.79 (2.07, 22.26)**	**4.71 (4.42, 5.02)**
**Age-Cohort for men**					
45–59 (Middle Age)	88.0%	35.4%	31.1%	2.33%	16.6
30–44 (Adult)	91.4%	37.7%	40.4%	1.79%	2.1
Ratio** (45–59/30–44)	**0.96 (0.92, 1.00)**	**0.94 (0.79, 1.12)**	**0.77 (0.63, 0.95)**	**1.30 (0.40, 4.24)**	**8.07 (7.22, 9.03)**
**Age-Cohort for women**					
45–59 (Middle Age)	81.6%	15.5%	21.8%	0.50%	3.4
30–44 (Adult)	89.1%	19.0%	36.5%	0.07%	0.6
Ratio** (45–59/30–44)	**0.92 (0.88, 0.96)**	**0.82 (0.59, 1.13)**	**0.60 (0.46, 0.78)**	**6.76 (0.42, 109.29)**	**5.67 (4.52, 6.91)**
**Race for men**					
White	90.8%	38.8%	43.5%	2.37%	11.0
Black	76.5%	20.7%	17.6%	1.88%	9.2
Ratio** (White/Black)	**1.19 (1.11, 1.27)**	**1.88 (1.43, 2.47)**	**2.47 (1.82, 3.36)**	**1.26 (0.56, 2.85)**	**1.20 (1.10, 1.32)**
**Race for women**					
White	90.7%	22.4%	40.5%	0.40%	2.2
Black	63.2%	8.2%	12.7%	0.00%	2.6
Ratio** (White/Black)	**1.44 (1.29, 1.60)**	**2.74 (1.60, 4.70)**	**3.18 (2.40, 4.22)**	**undefined**	**0.85 (0.73, 1.01)**

* These categories do not include data on 60–69 year old individuals since this data was not collected in that age group.

** Prevalence ratio for NHANES data, incidence rate ratio for SEER data.

While prevalence of ever oral sex was similar in men and women (PR = 1.03), men were ∼seven times more likely than women to have oral HPV16 infection and ∼five times as likely as women to be diagnosed with OSCC (Table 4). Number of oral sex partners was higher in men than women (PR for>5 lifetime oral sex partners = 1.8; PR for >10 lifetime oral sex partners = 3.0), but these behavioral ratios were notably smaller than those for oral HPV16 prevalence and OSCC by gender.

When examining prevalence ratios by age-cohort, older men were moderately less likely to report oral sexual behaviors (PRs 0.77–0.96), but had non-significantly higher oral HPV16 prevalence (PR = 1.3). As expected, OSCC rates increased dramatically with older age (IRR = 8.1, Table 4). Similarly, among women, older individuals were less likely to report sexual behaviors of interest (PRs<1.0), yet were ∼5-times more likely to have an oral HPV16 infection or OSCC.

When comparing white and black men, differences in sexual behaviors were consistent with the differences observed in oral HPV16 infection and cancer. Prevalence ratios for ever performing oral sex and >5 lifetime oral sex partners were 1.2 and 1.9, respectively for white compared to black men. There was a similarly modest increase in oral HPV16 infection (PR = 1.3) and OSCC (IRR = 1.2) among white compared to black men (Table 4).

### Multivariate Analysis of Risk Factors for Oral Sex

To further understand the contribution of these differences in oral sexual behaviors to the demographic differences in oral HPV16 prevalence, multivariate analyses were performed. We first evaluated the association between demographic characteristics of interest and oral sexual behaviors (Table 5). While male gender was not associated with ever having performed oral sex, men had on average five more oral sex partners than women (β = 5.23, 95%CI 1.91–8.56). Younger age-cohort and white race were each independently associated with increased odds of ever having oral sex (Table 5). Indeed, compared to 60–69 yo the odds of ever oral sex were significantly increased among 45–59 yo (aOR = 3.16 95%CI = 2.31–4.32) and 30–44 yo (aOR = 7.23 95%CI = 4.83–10.82). Similarly, whites had higher odds of ever oral sex than blacks and on average had 4 more oral sex partners when compared with blacks (β = 4.2, 95%CI = 1.75–6.6). Therefore, gender, age-cohort and race were each independent predictors of oral sexual behavior (Table 5).

**Table 5:** Multivariate risk factors among males and females associated with ever performing oral sex, number of lifetime oral sex partners*, oral HPV infection and oral HPV16 infection.

	Ever performed oral sex	Number of lifetime oral sex partners*	Oral HPV*	Oral HPV16*
	Odds Ratio(95% CI)	Slope (β, 95% CI)	Odds Ratio(95% CI)	Odds Ratio(95% CI)
**Gender**				
Female	1.00	1.00	1.00	1.00
Male	0.92 (0.75, 1.13)	5.23 (1.91, 8.56)	3.02 (2.08, 4.39)	5.01 (0.71, 35.26)
**Age**				
60–69 years	1.00	–	–	–
45–59 years	3.16 (2.31, 4.32)	0.00	1.00	1.00
30–44 years	7.23 (4.83, 10.82)	0.81(−3.58, 5.20)	0.68 (0.37, 1.24)	0.63 (0.18, 2.22)
20–29 years	5.82 (3.78, 8.98)	−2.80 (−6.50, 0.90)	0.73 (0.44, 1.23)	0.82 (0.14, 4.75)
**Race**				
Black	1.00	0.00	1.00	1.00
White	3.57 (2.55, 5.00)	4.17 (1.75, 6.60)	0.46 (0.29, 0.72)	1.46 (0.47, 4.51)
Mexican-American	0.78 (0.55, 1.12)	−0.61 (−1.89, 0.68)	0.55 (0.29, 1.03)	0.55 (0.17, 1.85) *(Other* ∧ *)*
Hispanic	1.65 (0.91. 2.98)	0.77 (−0.61, 2.15)	0.59 (0.32, 1.06)	
Other/Multi	0.72 (0.32, 1.63)	4.04 (−5.30, 13.39)	0.59 (0.24, 1.44)	
**Any college education**				
No	1.00	–	–	–
Yes	2.08 (1.77, 2.44)	–	–	–
**Ever married**				
No	1.00	–	–	–
Yes	2.99 (2.10, 4.27)	–	–	–
**Ever smoked**				
No	1.00	1.00	1.00	
Yes	1.85 (1.48, 2.31)	5.82 (1.49, 10.16)	1.66 (1.14, 2.41)	
**Ever drank alcohol regularly**				
No	1.00	–	–	–
Yes	3.12 (2.43, 4.01)	–	–	–
**Number of people performed oral sex on in lifetime** *				
0	–	–	1.00	1.00
1–2	–	–	0.79 (0.36, 1.76)	0.32 (0.04, 2.39)
3–5	–	–	1.69 (0.96, 2.96)	0.61 (0.08, 4.74)
6–10	–	–	2.12 (0.97, 4.64)	1.10 (0.11, 10.66)
>10	–	–	3.65 (1.52, 8.75)	3.88 (0.27, 55.37)
p-trend	–	–	p<0.001	p = 0.03

* These analyses do not include data on 60–69 year old since number of lifetime oral sex partners were not collected in that age group.

∧ Mexican-American, Hispanic any race and other/multi-race individuals were combined into a single “other” race category for this model of predictors of oral HPV16 because of low numbers of oral HPV16 infections in these groups.

By contrast, when evaluating the association between these demographic characteristics and prevalent oral HPV, after accounting for oral sexual behavior, age-cohort and white race were not associated with increased odds of oral HPV16 or any oral HPV (Table 5). Indeed, in multivariate analysis, only number of oral sexual partners (p-trend = 0.03) and gender (aOR 5.0, 95%CI 0.71–35.3) were associated with increased odds of prevalent oral HPV16. Similar results were observed when examining predictors of any oral HPV infection (Table 5). Results of these multivariate analyses were similar when stratified by gender and including all individuals 20–69 and adjusting for ever oral sex (Table S3 in File S1).

## Discussion

This study describes gender, age-cohort, and racial/ethnic differences in oral sexual practices in a representative sample of the U.S population. These data reveal that there are differences in oral sexual behaviors when considering gender, age-cohort and race. Men, younger age-cohorts, and whites have higher exposures to the oral sexual behaviors associated with oral HPV16 infection and OSCC. [12], [13], [26] In multivariate analysis, oral sexual behavior and gender are each associated with the presence of an oral HPV16 infection, while age-cohort and race are not. This suggests that the observed epidemiologic differences in infection are a result of differences in oral sexual behavioral, and not by age-cohort or race differences. This study is the first to explore whether differences in sexual behaviors across gender, age-cohort, and race account for differences in oral HPV16 infection and OSCC in these same groups.

The relative differences in prevalence of oral sexual behaviors, oral HPV16 infection, and OSCC incidence are dramatic when considering gender (Table 4). Prevalence of oral HPV16 and incidence of OSCC are each 5–7 fold higher in men than women. Males, independent of age and race, have more oral sexual partners as compared with females. Indeed, the incidence of HPV-OSCC has increased by 4-fold in men over the past 20 years, [2] while the proportion of men ever performing oral sex in these age-cohorts has increased more modestly from 73% to 90%. Further, male gender is associated with oral HPV16 prevalence, even after accounting for oral sexual behavior. This suggests that in addition to the behavioral differences between men and women, namely higher cumulative exposure to oral HPV16 infection, additional gender-specific factors may account for the increased prevalence of oral HPV16 infection or incidence of OSCC in men compared to women. A potential explanation for male gender being an independent risk factor for oral HPV16 infection is that performing oral sex on a woman might have higher infection risk than performing oral sex on a man. [27] While there was a suggestion that oral HPV prevalence might be higher among lesbian than heterosexual women in this data, there were a limited number of lesbian/bisexual women in this study and this difference was not statistically significant. Genetic and immune differences may also contribute to the gender differences in oral HPV infection and HPV-OSCC.

Notable age-cohort differences in oral sexual behaviors were observed. Our finding that oral sex was significantly more common among young adults than older adults is consistent with prior data. In recent decades, the age of sexual debut has decreased and the number of lifetime sexual partners has increased. [6], [11], [12] Although younger age-cohorts are more likely to report oral sexual behavior, age-cohort had no independent effect on the odds of oral HPV16 infection in this study. It appears that oral HPV16 infection varies as a function of oral sexual behaviors. While these oral sexual behaviors differ significantly by age-cohort, age does not appear to *independently* influence the presence of oral HPV16 infection (i.e. the differences in oral HPV are explained by differences in behaviors between the age-cohorts). This finding may be due to the age-categorization used, as the presence of a bimodal relationship of age and oral HPV prevalence has been suggested when this NHANES data was modeled. [22] We extrapolate from these data that these behavioral differences by age-cohort may similarly contribute to the observed increase in HPV-OSCC incidence. This is consistent with the hypothesis that changes in sexual behavior explain the increase in HPV-OSCC. However, it is important to recognize that cancers across anatomic sites increase with age, and that this question was not directly examined in this dataset.

This study also describes differences in oral sexual behaviors among blacks and whites in the U.S. Whites were significantly more likely than other races to report oral sexual behaviors, even after accounting for other important risk factors including age and gender. Yet, white race was not independently associated with oral HPV16 after adjusting for oral sexual behavior. Similar to age-cohort differences in oral sexual behavior, racial differences in oral sexual behaviors explain the racial disparities observed in unadjusted oral HPV16 prevalence and possibly HPV-OSCC incidence (although this was not assessed in this study). Interestingly, while oral HPV16 infection was *less* common in blacks than whites, prevalence of any oral HPV was significantly *more* common in blacks than whites; it is unclear why this difference was observed. Given that tumor HPV status for SEER OSCCs is unknown, the ratio of HPV-OSCC comparing whites to blacks may be higher than our estimated incidence rate ratio for OSCC (Table 4). However, it remains unlikely that these relative differences would be larger than the 1.9–2.5-fold higher behavioral differences observed in white compared to black men for high number of oral sex partners and oral sex around sexual debut. The racial differences in oral sex described in this study of individuals ≥20 yo, are consistent with another study of 15–19 yo, in which white males were three times more likely to engage in oral sex than black males [28] and whites were more likely to first perform oral sex at a younger age. A substantial difference in the ordering of sexual acts at sexual debut among blacks and whites was observed for both genders (PR male = 2.5, female = 3.2). The racial differences in first sexual act observed are similar to a study of 15–24 yo, where 27% of non-Hispanic black compared to 49% of non-Hispanic white youths performed oral sex as their first sexual act [14].

In our analysis, we show that oral sexual behaviors vary by gender, age-cohort and race. We also describe similar relative differences in prevalence of oral HPV16 infection and incidence of HPV-OSCC. However, after accounting for oral sexual behavior, we learn that the age and race do not independently affect the odds of oral HPV16 infection. Instead, sexual behavior appears to be driving the prevalence of oral HPV16 infection and therefore, is likely to have a similar effect on HPV-OSCC (although not analyzed here). After controlling for number of oral sexual partners, gender remained associated with oral HPV prevalence, and was elevated but not statistically significantly associated with oral HPV16 prevalence; this suggests that while number of oral sexual partners may be the primary risk factor for oral HPV infection there may be other sexual factors (such as performing oral sex on a women compared to a man, or site of first HPV exposure (genital vs. oral), hormonal factors (sex steroids), or other unknown factors which also influence the observed gender difference in oral HPV prevalence. While this study suggests that changes in oral sexual behavior likely contribute to the observed increases in HPV-OSCC, there may be other unknown factors that may also contribute to these epidemiologic differences.

This study has several limitations and strengths. Study limitations include the lack of some sexual data in 60–69 yo, the lack of temporal data, and the limited number of individuals with oral HPV16 infection. The NHANES and SEER data are not linked, and HPV tumor status of OSCC in SEER is unknown. However, since ∼70% of current OSCCs are HPV-positive, [3] the IRRs for HPV-OSCC may be higher. Strengths of this study include the large overall sample size with a wide range of ages and significant representation of blacks and other minority populations, as well as the availability of oral HPV prevalence data. NHANES data are representative of the U.S. population. SEER data provides population based cancer incidence estimates. These data are a significant contribution to the small but growing body of literature which examines the contribution of HPV to temporal changes in HPV-OSCC and the role of gender and race in these cancers.

This data provide an important context regarding changing sexual behaviors and the role these behaviors may play in the increasing incidence of HPV-OSCC. We learn from this data that oral sexual behaviors are common and that males, younger age groups, and whites report increased oral sexual behaviors, and thus likely have a higher exposure to oral HPV infections. However, age-cohort and race are not independent predictors of oral HPV infections, but rather affect the odds of oral HPV infection through differences in oral sexual behaviors. Similar to U.S. epidemiologic trends, OSCC appears to be rising among younger men in some economically developed countries, while incidence is decreasing or stable OSCC in other regions. [29] As the data presented in this paper relate only to U.S. sexual behaviors, additional research will be needed to understand whether similar changes in oral sexual behavior are occurring in other regions with increasing OSCC incidence.

Recent research suggests HPV vaccination is likely effective in preventing oral HPV infection among women, with efficacy expected among men as well. [30] As this study informs us that sexual behaviors and male gender are independently associated with oral HPV infection, the cause of HPV-OSCC, it underscores the need for early HPV vaccination among not only girls, but also among boys.

## Supporting Information

File S1 **Table S1. Characteristics of the 4256 individuals in NHANES study population contributing data to this analysis. Table S2. Sexual behavior and oral HPV prevalence by age-cohorts. Table S3. Multivariate risk factors associated with ever oral sex, oral HPV infection, when controlling for ever performing oral sex, among 20–69 year olds, and stratified by gender.**
(DOCX)

## References

[1] D’Souza G, Kreimer AR, Viscidi R, Pawlita M, Fakhry C, et al (2007) Case-control study of human papillomavirus and oropharyngeal cancer. N Engl J Med 356: 1944–1956. 17494927 10.1056/NEJMoa065497

[2] Chaturvedi AK, Engels EA, Anderson WF, Gillison ML (2008) Incidence trends for human papillomavirus-related and -unrelated oral squamous cell carcinomas in the United States. J Clin Oncol 26: 612–619. 18235120 10.1200/JCO.2007.14.1713

[3] Chaturvedi A, Engels E, Pfeiffer R, Hernandez BY, Xiao W, et al (2011) Human papillomavirus (HPV) and rising oropharyngeal cancer incidence in the United States. J Clin Oncol 29: 4294–4301. 21969503 10.1200/JCO.2011.36.4596 PMC3221528

[4] Jemal A, Thun MJ, Ries LA, Howe HL, Weir HK, et al (2008) Annual report to the nation on the status of cancer, 1975–2005, featuring trends in lung cancer, tobacco use, and tobacco control. J Natl Cancer Inst 100: 1672–1694. 19033571 10.1093/jnci/djn389 PMC2639291

[5] Ramqvist T, Dalianis T (2010) Oropharyngeal cancer epidemic and human papillomavirus. Emerging infectious diseases 16: 1671–1677. 21029523 10.3201/eid1611.100452 PMC3294514

[6] Bajos N, Bozon M, Beltzer N, Laborde C, Andro A, et al (2010) Changes in sexual behaviours: from secular trends to public health policies. AIDS 24: 1185–1191. 20299962 10.1097/QAD.0b013e328336ad52

[7] Agius PA, Pitts MK, Smith AM, Mitchell A (2010) Sexual behaviour and related knowledge among a representative sample of secondary school students between 1997 and 2008. Australian and New Zealand Journal of Public Health 34: 476–481. 21040175 10.1111/j.1753-6405.2010.00593.x

[8] Herlitz C (2009) Sexual risk-taking in the general population of Sweden (1989–2007). Sexual health 6: 272–280. 19917194 10.1071/SH08095

[9] Satterwhite CL, Kamb ML, Metcalf C, Douglas JM Jr, Malotte CK, et al (2007) Changes in sexual behavior and STD prevalence among heterosexual STD clinic attendees: 1993–1995 versus 1999–2000. Sexually transmitted diseases 34: 815–819. 17551414 10.1097/OLQ.0b013e31805c751d

[10] Aral SO, Patel DA, Holmes KK, Foxman B (2005) Temporal trends in sexual behaviors and sexually transmitted disease history among 18- to 39-year-old Seattle, Washington, residents: results of random digit-dial surveys. Sexually transmitted diseases 32: 710–717. 16254547 10.1097/01.olq.0000175370.08709.72

[11] Turner CF, Danella RD, Rogers SM (1995) Sexual behavior in the United States 1930–1990: trends and methodological problems. Sexually transmitted diseases 22: 173–190. 7652662 10.1097/00007435-199505000-00009

[12] Herbenick D, Reece M, Schick V, Sanders SA, Dodge B, et al (2010) Sexual behavior in the United States: results from a national probability sample of men and women ages 14–94. The journal of sexual medicine 7 Suppl 5255–265. 21029383 10.1111/j.1743-6109.2010.02012.x

[13] Leichliter JS, Chandra A, Liddon N, Fenton KA, Aral SO (2007) Prevalence and correlates of heterosexual anal and oral sex in adolescents and adults in the United States. J Infect Dis 196: 1852–1859. 18190267 10.1086/522867

[14] Copen CE, Chandra A, Martinez G (2012) Prevalence and timing of oral sex with opposite-sex partners among females and males ages 15–24: United States, 2007–2010. U.S. Department of Health and Human Services. 24979976

[15] Ang K, Harris J, Wheeler R, Weber R, Rosenthal D, et al (2010) Human Papillomavirus and Survival of Patients with Oropharyngeal Cancer. N Engl J Med 363: 24–35. 20530316 10.1056/NEJMoa0912217 PMC2943767

[16] Weinberger PM, Merkley MA, Khichi SS, Lee JR, Psyrri A, et al (2010) Human papillomavirus-active head and neck cancer and ethnic health disparities. Laryngoscope 120: 1531–1537. 20564751 10.1002/lary.20984 PMC3051373

[17] Fakhry C, Westra WH, Li S, Cmelak A, Ridge JA, et al (2008) Improved survival of patients with human papillomavirus-positive head and neck squamous cell carcinoma in a prospective clinical trial. J Natl Cancer Inst 100: 261–269. 18270337 10.1093/jnci/djn011

[18] Gillison ML, D’Souza G, Westra W, Sugar E, Xiao W, et al (2008) Distinct risk factor profiles for human papillomavirus type 16-positive and human papillomavirus type 16-negative head and neck cancers. J Natl Cancer Inst 100: 407–420. 18334711 10.1093/jnci/djn025

[19] Settle K, Posner MR, Schumaker LM, Tan M, Suntharalingam M, et al (2009) Racial survival disparity in head and neck cancer results from low prevalence of human papillomavirus infection in black oropharyngeal cancer patients. Cancer prevention research (Philadelphia, Pa) 2: 776–781. 10.1158/1940-6207.CAPR-09-0149 PMC4459126 19641042

[20] Cole L, Polfus L, Peters ES (2012) Examining the Incidence of Human Papillomavirus-Associated Head and Neck Cancers by Race and Ethnicity in the U.S. 1995–2005. PLoS ONE 7: e32657. 22448226 10.1371/journal.pone.0032657 PMC3308956

[21] Jemal A, Simard EP, Dorell C, Noone AM, Markowitz LE, et al (2013) Annual Report to the Nation on the Status of Cancer, 1975–2009, Featuring the Burden and Trends in Human Papillomavirus (HPV)-Associated Cancers and HPV Vaccination Coverage Levels. J Natl Cancer Inst 105: 175–201. 23297039 10.1093/jnci/djs491 PMC3565628

[22] Gillison ML, Broutian T, Pickard RK, Tong ZY, Xiao W, et al (2012) Prevalence of oral HPV infection in the United States, 2009–2010. Jama 307: 693–703. 22282321 10.1001/jama.2012.101 PMC5790188

[23] NHANES 2009–10 National Health and Nutrition Examination Survey Data Centers for Disease Control and Prevention (CDC) National Center for Health Statistics (NCHS). Hyattsville, MD: U.S. Department of Health and Human Services, Centers for Disease Control and Prevention.

[24] Broutian TR, He X, Gillison ML (2010) Automated high throughput DNA isolation for detection of human papillomavirus in oral rinse samples. Journal of clinical virology : the official publication of the Pan American Society for Clinical Virology. 10.1016/j.jcv.2010.12.005 PMC3059388 21273118

[25] Surveillance E, and End Results (SEER) Program (2013) SEER*Stat Database: Incidence - SEER 9 Regs Research Data, Nov 2011 Sub, Vintage 2009 Pops (1973–2009), National Cancer Institute, DCCPS, Surveillance Research Program, Surveillance Systems Branch, released April 2012, based on the November 2011 submission.

[26] Ryerson AB, Peters ES, Coughlin SS, Chen VW, Gillison ML, et al (2008) Burden of potentially human papillomavirus-associated cancers of the oropharynx and oral cavity in the US, 1998–2003. Cancer 113: 2901–2909. 18980273 10.1002/cncr.23745

[27] Tobian AA, Kong X, Gravitt PE, Eaton KP, Kigozi G, et al (2011) Male circumcision and anatomic sites of penile high-risk human papillomavirus in Rakai, Uganda. Int J Cancer 129: 2970–2975. 21462185 10.1002/ijc.25957 PMC3193547

[28] Gates GJ, Sonenstein FL (2000) Heterosexual genital sexual activity among adolescent males: 1988 and 1995. Family planning perspectives 32: 295–297, 304. 11138866

[29] Chaturvedi AK, Anderson WF, Lortet-Tieulent J, Curado MP, Ferlay J, et al.. (2013) Worldwide Trends in Incidence Rates for Oral Cavity and Oropharyngeal Cancers. J Clin Oncol. 10.1200/JCO.2013.50.3870 PMC3865341 24248688

[30] Herrero R, Quint W, Hildesheim A, Gonzalez P, Struijk L, et al (2013) Reduced Prevalence of Oral Human Papillomavirus (HPV) 4 Years after Bivalent HPV Vaccination in a Randomized Clinical Trial in Costa Rica. PloS one 8: e68329. 23873171 10.1371/journal.pone.0068329 PMC3714284

