# Supplementary material for: Differences in Oral Sexual Behaviors by Gender, Age, and Race Explain Observed Differences in Prevalence of Oral Human Papillomavirus Infection
Source: PLoS One. 2014 Jan 24;9(1):e86023. doi: 10.1371/journal.pone.0086023 (PMC3901667; doi:10.1371/journal.pone.0086023)
Supplement: File S1 — Table S1. Characteristics of the 4256 individuals in NHANES study population contributing data to this analysis. Table S2. Sexual behavior and oral HPV prevalence by age-cohorts. Table S3. Multivariate risk factors associated with ever oral sex, oral HPV infection, when controlling for ever performing oral sex, among 20–69 year olds, and stratified by gender. (DOCX) [file pone.0086023.s001.docx]

**Supplemental Table S1. Characteristics of the 4256 individuals in NHANES study population contributing data to this analysis**

|  | **Sampled Individuals** | | **Weighted U.S. prevalence** |
| --- | --- | --- | --- |
|  | **N** | **%** |  |
| **Gender** |  |  |  |
| Male | 2,116 | 49.7% | 50.4% |
| Female | 2,140 | 50.3% | 49.6% |
| **Age Cohort** |  |  |  |
| 20-29 (young adult) | 887 | 20.8% | 21.6% |
| 30-44 (adult) | 1,299 | 30.5% | 31.2% |
| 45-59 (middle age) | 1,276 | 30.0% | 32.7% |
| 60-69 (senior) | 794 | 18.7% | 14.5% |
| **Race** |  |  |  |
| White non-Hispanic | 1,942 | 45.6% | 68.0% |
| Black non-Hispanic | 781 | 18.4% | 11.3% |
| Mexican-American | 837 | 19.7% | 9.0% |
| Any race, Hispanic | 482 | 11.3% | 5.4% |
| Other race, non-Hispanic/ multi-racial | 214 | 5.0% | 6.4% |
| **Cigarette Use** |  |  |  |
| Never smoker | 2,367 | 55.6% | 57.4% |
| Former smoker | 847 | 19.9% | 21.0% |
| Current smoker | 1,042 | 24.5% | 21.6% |
| **Number of people in household** |  |  |  |
| 1 | 450 | 10.6% | 10.9% |
| 2 | 1,114 | 26.2% | 30.9% |
| 3 | 761 | 17.9% | 18.7% |
| 4 | 840 | 19.7% | 20.3% |
| ≥5 | 1,091 | 25.6% | 19.0% |
| **Marital Status** |  |  |  |
| Married | 2,187 | 51.4% | 56.2% |
| Widowed | 131 | 3.1% | 2.3% |
| Divorced or Separated | 656 | 15.4% | 13.1% |
| Never Married | 854 | 20.1% | 19.6% |
| Living With Partner | 424 | 10.0% | 8.8% |
| **Education** |  |  |  |
| <9^th^ grade | 433 | 10.2% | 4.9% |
| 9-11 grade | 652 | 15.3% | 11.5% |
| High school graduate / GED | 993 | 23.4% | 22.8% |
| Some college or AA degree | 1,265 | 29.8% | 31.5% |
| College graduate+ | 906 | 21.3% | 29.2% |
| **Sexual Orientation (20-59 yo)*** |  |  |  |
| Heterosexual | 3,203 | 92.5% | 93.8% |
| Homosexual | 44 | 1.3% | 1.4% |
| Bi-sexual / something else | 126 | 3.6% | 3.1% |
| Not reported/ unknown | 89 | 2.6% | 1.8% |
| **In lifetime, more than five:** |  |  |  |
| Sexual partners | 2,122 | 50.3% | 50.4% |
| Oral sex partners (20-59 yo)* | 771 | 22.4% | 25.1% |

* These variables were not collected on 60-69 year old individuals

**Supplemental Table S2. Sexual behavior and oral HPV prevalence by age-cohorts**

|  | **20-29** | **30-44** | **45-59** | **60-69** |  |
| --- | --- | --- | --- | --- | --- |
|  | **(young adult)** | **(adult)** | **(middle age)** | **(senior)** |  |
|  | **N=887** | **N=1299** | **N=1276** | **N=794** | **P-value** |
| **Number of lifetime sexual partners:** |  |  |  |  |  |
| Any sexual act: median | 5 | 6 | 6 | 4 | --- |
| Any sexual act: mean | 9.5 | 14.0 | 14.8 | 13.2 | <0.001 |
| Performed oral sex on*: median | 2 | 3 | 2 | --- | --- |
| Performed oral sex on*: mean | 4.4 | 7.4 | 8.1 | --- | <0.001 |
| **Age at first:** |  |  |  |  |  |
| Sexual act: median | 16 | 17 | 17 | 18 | --- |
| Sexual act: mean | 16.4 | 17.3 | 17.8 | 18.8 | <0.001 |
| Performed oral sex on*: median | 17 | 19 | 20 | --- | --- |
| Performed oral sex on*: mean | 17.6 | 19.8 | 21.3 | --- | <0.001 |
| **Number of lifetime sexual partners** |  |  |  |  | <0.001 |
| 0 | 8.9% | 1.5% | 1.9% | 2.4% |  |
| 1-2 | 20.3% | 18.7% | 20.0% | 33.0% |  |
| 3-5 | 22.9% | 23.1% | 27.2% | 25.3% |  |
| 6-10 | 23.9% | 26.6% | 22.1% | 16.3% |  |
| >10 | 24.0% | 30.2% | 28.8% | 23.1% |  |
| **Ever performed oral sex** |  |  |  |  | <0.001 |
| No | 17.3% | 9.7% | 15.2% | 27.3% |  |
| Yes | 82.7% | 90.3% | 84.8% | 72.7% |  |
| **Number of people performed oral sex on in lifetime*** |  |  |  |  | 0.005 |
| 0 | 17.4% | 9.8% | 15.3% | --- |  |
| 1-2 | 34.8% | 36.2% | 37.1% | --- |  |
| 3-5 | 28.2% | 25.5% | 22.1% | --- |  |
| 6-10 | 11.0% | 14.2% | 13.8% | --- |  |
| >10 | 8.6% | 14.3% | 11.7% | --- |  |
| **Comparison of number of lifetime oral and vaginal sexual partners*** |  |  |  |  | 0.078 |
| Fewer oral sex partners | 59.2% | 65.1% | 67.9% | --- |  |
| Same # oral & vaginal | 26.9% | 24.5% | 23.7% | --- |  |
| More oral sex partners | 13.8% | 10.4% | 8.4% | --- |  |
| **First sexual experience** <18 years | 63.0% | 61.4% | 51.1% | 37.3% | <0.001 |
| **First performed oral sex*** <18 years | 42.9% | 30.9% | 19.5% | --- | <0.001 |
| **Age at first oral sex relative to sexual debut*** |  |  |  |  | <0.001 |
| Oral sex at sexual debut | 44.4% | 38.5% | 26.5% | --- |  |
| Oral sex after sexual debut | 38.3% | 51.7% | 58.2% | --- |  |
| Never oral sex | 17.4% | 9.7% | 15.3% | --- |  |
| **Oral HPV Prevalence** | N=864 | N=1228 | N=1208 | N=759 |  |
| HPV16 | 0.9% | 0.9% | 1.4% | 1.3% | 0.860 |
| Any HPV type | 6.2% | 6.4% | 8.7% | 8.3% | 0.344 |

*These categories do not include data on 60-69 year old individuals since this data was not collected in that age group

**Supplemental Table S3**. **Multivariate risk factors associated with ever oral sex, oral HPV infection, when controlling for ever performing oral sex, among 20-69 year olds, and stratified by gender**

|  | **Odds Ratio (95% CI)** | | | | | |
| --- | --- | --- | --- | --- | --- | --- |
|  | **Males** | | | **Females** | | |
|  | **Ever performed oral sex** | **Oral HPV** | **Oral HPV16** | **Ever performed oral sex** | **Oral HPV** | **Oral HPV16** |
| **Race** |  |  |  |  |  |  |
| Black | 1.00 | 1.00 | 1.00 | 1.00 | 1.00 | 1.00 ***[****All non-white]* |
| White | 2.49 (1.73, 3.59) | 0.42 (0.28, 0.64) | 1.19 (0.49, 2.90) | 5.01 (3.34, 7.53) | 0.85 (0.32, 2.26) | 4.04 (0.39, 41.41) |
| Mexican-American | 0.53 (0.31, 0.90) | 0.37 (0.22, 0.64) | 0.38 (0.14, 1.01) ^ | 1.07 (0.72, 1.59) | 1.50 (0.49, 4.66) | -- |
| Other Hispanic | 1.53 (0.74, 3.16) | 0.46 (0.25, 0.84) |  | 1.79 (0.95, 3.38) | 1.58 (0.70, 3.57) | -- |
| Other/Multi | 0.36 (0.13, 0.98) | 0.48 (0.16, 1.41) |  | 1.33 (0.53, 3.32) | 0.68 (0.13, 3.64) | -- |
| **Age** |  |  |  |  |  |  |
| 60-69 years | 1.00 | 1.00 | 1.00 | 1.00 | 1.00 | 1.00 |
| 45-59 years | 4.26 (2.86, 6.37) | 0.99 (0.62, 1.59) | 1.10 (0.27, 4.42) | 2.42 (1.47, 4.01) | 0.93 (0.37, 2.39) | 2.05 (0.11, 39.41) |
| 30-44 years | 8.13 (4.92, 13.42) | 0.76 (0.48, 1.18) | 0.87 (0.25, 3.04) [*20-44]* | 6.38 (3.72, 10.96) | 0.53 (0.15, 1.83) | 0.33 (0.01, 8.64) *[20-44]* |
| 20-29 years | 6.61 (3.74, 11.66) | 0.60 (0.31, 1.19) |  | 5.61 (3.07, 10.23) | 0.72 (0.26, 2.00) |  |
| **Any college education** |  |  |  |  |  |  |
| No | 1.00 | -- | -- | 1.00 | -- | -- |
| Yes | 1.84 (1.28, 2.64) | -- | -- | 2.38 (1.75, 3.23) | -- | -- |
| **Ever married** |  |  |  |  |  |  |
| No | 1.00 | -- | -- | 1.00 | 1.00 | -- |
| Yes | 4.24 (2.66, 6.79) | -- | -- | 2.03 (1.43, 2.88) | 0.46 (0.26, 0.82) | -- |
| **Ever smoked** |  |  |  |  |  |  |
| No | 1.00 | 1.00 | -- | 1.00 | 1.00 | -- |
| Yes | 1.67 (1.16, 2.41) | 1.83 (1.23, 2.72) | -- | 2.08 (1.34, 3.23) | 2.12 (1.01, 4.44) | -- |
| **Ever drank alcohol regularly** |  |  |  |  |  |  |
| No | 1.00 | -- | -- | 1.00 | -- | -- |
| Yes | 4.05 (2.85, 5.76) | -- | -- | 2.56 (1.81, 3.62) | -- | -- |
| **Ever performed oral sex** |  |  |  |  |  |  |
| No | -- | 1.00 | 1.00 | -- | 1.00 | 1.00 |
| Yes | -- | 1.78 (0.78, 4.03) | 1.40 (0.16, 11.82) | -- | 1.93 (1.09, 3.41) | 61.84 (3.93, 973.75)******* |

^ Mexican-American, Hispanic any race and other/multi-race individuals were combined into a single “other” race category for this model of predictors of oral HPV16 because of low numbers of oral HPV16 infections in these groups

≤ 10 lifetime oral sex partners used as the reference since there were no oral HPV16 infections among those who never performed oral sex. For 60-69 year olds who ever performed oral sex, the number of oral sex partners was imputed as the number of total sex partners
